# PUBLISHING WITH THE INTERNATIONAL JOURNAL OF AGING AND HUMAN DEVELOPMENT

**DOI:** 10.1093/geroni/igad104.0953

**Published:** 2023-12-21

**Authors:** Julie Hicks Patrick

**Affiliations:** West Virginia University, Morgantown, West Virginia, United States

## Abstract

For more than 50 years, under the leadership of four editors and two publishers, The International Journal of Aging and Human Development (IJAHD) has featured multidisciplinary scholarship related to aging processes and older adults. With the publication of eight issues a year and over 1000 pages of scientific content, the IJAHD places emphasis upon psychological and social studies of aging and the aged. However, the Journal also publishes research that integrates observations from other disciplines that illuminate the “human” side of gerontology. A more recent focus includes midlife development, as well. About half (47%) of the publications in the IJAHD are from international colleagues. IJAHD is delighted to support new investigators as they navigate dissemination. This presentation will discuss tips for both international and US-based scholars for ensuring timely reviews and positive decisions for manuscript submissions, including such areas as key words, suggesting unbiased reviewers, formatting, writing mechanics, clearly-articulated methods, and a sound theoretical basis.

